# Monographs for medicines on WHO’s Model List of Essential Medicines

**DOI:** 10.2471/BLT.17.205807

**Published:** 2018-03-28

**Authors:** Lukas Roth, Melissa Adler, Tanvi Jain, Daniel Bempong

**Affiliations:** a Global Public Health Division, United States Pharmacopeia, 12601 Twinbrook Parkway, Rockville, Maryland 20852, United States of America.

## Abstract

**Objective:**

To raise awareness about the importance of public pharmaceutical standards, identify if and, if so, where current pharmacopeias are falling short in the development of new and complete monographs and foster collaboration among the various pharmacopeias, to prioritize, develop and make available standards for those key medicines for which no complete monographs exist.

**Methods:**

In August 2017, we mined eight pharmacopeias to identify which of the 669 medicines in the 20th edition of the World Health Organization’s Model List of Essential Medicines were covered by complete or incomplete monographs. The pharmacopeias we included were the Brazilian Pharmacopoeia, the British Pharmacopoeia, the Indian Pharmacopeia Commission, the International Pharmacopoeia, the Japanese Pharmacopoeia, the Mexican Pharmacopoeia, the Pharmacopeia of the People’s Republic of China and the United States Pharmacopeia.

**Findings:**

For 99 (15%) of the medicines on the Model List, no monographs were available in any of the eight pharmacopeias investigated. Only 3% (1/30) of the cardiovascular medicines listed, but 28% (9/32) of the antiretroviral medicines and 23% (6/26) of the antimalarial medicines lacked monographs.

**Conclusion:**

There appear to be no public standards for many so-called essential medicines. To address this shortfall, a greater collaboration in the global health community is needed.

## Résumé

**Objectif:**

Sensibiliser à l'importance des normes pharmaceutiques publiques, déterminer si certaines pharmacopées actuelles présentent des lacunes concernant l'élaboration de nouvelles monographies complètes et, si tel est le cas, lesquelles, et encourager la collaboration entre les différentes pharmacopées pour hiérarchiser, élaborer et publier des normes à l'égard des médicaments essentiels pour lesquels il n'existe aucune monographie complète.

**Méthodes:**

En août 2017, nous avons analysé huit pharmacopées pour déterminer quels médicaments parmi les 669 inclus dans la 20e édition de la Liste modèle de l'Organisation mondiale de la Santé des médicaments essentiels étaient couverts par des monographies complètes ou incomplètes. Les pharmacopées analysées étaient la Pharmacopée brésilienne, la Pharmacopée britannique, la Commission de la Pharmacopée indienne, la Pharmacopée internationale, la Pharmacopée japonaise, la Pharmacopée mexicaine, la Pharmacopée de la République populaire de Chine et la Pharmacopée des États-Unis.

**Résultats:**

Dans le cas de 99 (15%) médicaments inclus dans la Liste modèle, aucune monographie n'était disponible dans aucune des huit pharmacopées analysées. Seuls 3% (1/30) des médicaments cardiovasculaires inclus, mais 28% (9/32) des médicaments antirétroviraux et 23% (6/26) des médicaments antipaludiques n'étaient associés à aucune monographie.

**Conclusion:**

Il semblerait qu'il n'existe aucune norme publique pour de nombreux médicaments jugés essentiels. Afin de combler cette lacune, il est indispensable de renforcer la collaboration au sein de la communauté sanitaire mondiale.****

## Resumen

**Objetivo:**

Concienciar sobre la importancia de los estándares farmacéuticos públicos, identificar si, y, si es así, las farmacopeas actuales no son suficientes para el desarrollo de monografías nuevas y completas y fomentar la colaboración entre las distintas farmacopeas, para priorizar, desarrollar y poner a disposición estándares para los medicamentos clave para los cuales no existen monografías completas.

**Métodos:**

En agosto de 2017, se extrajeron ocho farmacopeas para identificar cuáles de los 669 medicamentos de la 20ª edición de la Lista Modelo de la Organización Mundial de la Salud (OMS) estaban cubiertos por monografías completas o incompletas. Las farmacopeas que incluimos fueron la Farmacopea Brasileña, la Farmacopea Británica, la Comisión de Farmacopea India, la Farmacopea Internacional, la Farmacopea Japonesa, la Farmacopea Mexicana, la Farmacopea de la República Popular de China y la Farmacopea de los Estados Unidos.

**Resultados:**

Para 99 (15 %) de los medicamentos en la Lista Modelo, no se encontraron monografías disponibles en ninguna de las ocho farmacopeas investigadas. Solo el 3 % (1/30) de los medicamentos cardiovasculares enumerados, mientras que el 28 % (9/32) de los medicamentos antirretrovirales y el 23 % (6/26) de los medicamentos antipalúdicos carecían de monografías.

**Conclusión:**

Parece que no existen estándares públicos para muchos de los considerados medicamentos esenciales. Para abordar este déficit, se necesita una mayor colaboración en la comunidad de la salud global.

## ملخص

**الغرض:**

رفع الوعي بأهمية المعايير الدوائية العامة، والوقوف على أي قصور – حال وجوده – في دساتير الأدوية الحالية من حيث تطوير دراسات متخصصة جديدة وكاملة، وتعزيز التعاون بين دساتير الأدوية المختلفة، وإعطاء الأولوية للمعايير وتطويرها وطرحها للأدوية الرئيسية التي تفتقر إلى دراسات متخصصة وكاملة.

**الطريقة:**

قمنا في شهر أغسطس/آب 2017 بفرز ثمانية دساتير للأدوية لمراجعة الأدوية البالغ عددها 669 دواءً في النسخة العشرين من قائمة الأدوية الأساسية النموذجية لمنظمة الصحة العالمية، وتحديد أي من تلك الأدوية قد تمت تغطيتها من خلال دراسات متخصصة كاملة أو غير كاملة. وكانت دساتير الأدوية التي قمنا بتضيمنها هي دستور الأدوية البرازيلي، ودستور الأدوية البريطاني، ولجنة دستور الأدوية الهندي، ودستور الأدوية الدولي، ودستور الأدوية الياباني، ودستور الأدوية المكسيكي، ودستور الأدوية المتبع في جمهورية الصين الشعبية، ودستور الأدوية الأمريكي.

**النتائج:**

بالنسبة إلى 99 دواءً واردًا في القائمة النموذجية (أي بواقع 15‏% منها)، لم تتوفر دراسات متخصصة في أي من دساتير الأدوية الثمانية التي شملها البحث. واقتصر العجز في الدراسات المتخصصة المتعلقة بأدوية القلب والأوعية الدموية على نسبة 3% (بواقع دواء واحد من بين 30 دواءً)، في حين ارتفعت النسبة إلى ‏28% (بواقع 9 من بين 32 دواءً) من الأدوية المضادة لفيروسات النسخ العكسي، و23‏% (بواقع 6 أدوية من بين 26 دواءً) من الأدوية المضادة للملاريا.

**الاستنتاج:**

لا يبدو أن هناك معايير عمومية لما يسمى بالأدوية النموذجية. ولكي يتم التعامل مع هذا العجز، فهناك حاجة لدرجة أكبر من التعاون في مجتمع الصحة العالمية.

## 摘要

**目的:**

旨在提高人们对公共药物标准重要性的意识，明确现有药典是否落后于最新完整专著的发展。如落后，明确具体落后在哪些方面，并促进不同药典之间的合作，从而优先考虑发展没有完整专著的关键药物，并为其制定标准。

**方法:**

2017 年 8 月，我们采纳了八部药典以明确第 20 版世界卫生组织《基本药物示范目录》 669 种药品中的哪些药物涵盖在完整或不完整专著中。我们纳入的药典包括《巴西药典》、《英国药典》、印度药典委员会出版的《印度药典》、《国际药典》、《日本药典》、《墨西哥药典》、《中华人民共和国药典》和《美国药典》。

**结果:**

在研究的八部药典中，共有 99 (15%) 种列于《基本药物示范目录》中的药物尚无专著。目录中的心血管药物只列出了 3% (1/30)，而 28% (9/32) 的抗逆转录病毒药物和 23% (6/26) 的抗疟药物缺少专著。

**结论:**

很多所谓的基本药物似乎并无公用的标准。全球卫生界需要加强合作以解决此方面的不足。****

## Резюме

**Цель:**

Повысить осведомленность о важности государственных фармацевтических стандартов, определить, где текущие фармакопеи не справляются с разработкой новых и полных монографий, и способствовать сотрудничеству между различными фармакопеями, чтобы определить приоритеты, разработать и предоставить доступные стандарты для ключевых лекарственных средств, не представленных полными монографиями.

**Методы:**

В августе 2017 года авторы сделали выборку из восьми фармакопей, чтобы определить, какие из 669 лекарственных средств в 20-м издании Примерного перечня основных лекарственных средств Всемирной организации здравоохранения были охвачены полными или неполными монографиями. В выборку вошли фармакопеи Бразилии, Индии, Китайской Народной Республики, Мексики, Соединенных Штатов Америки и Японии, а также Британская и Международная.

**Результаты:**

Для 99 лекарственных средств (15%), входящих в Типовой перечень, не было доступно никаких монографий ни в одной из восьми исследованных фармакопей. Только 3% перечисленных сердечно-сосудистых препаратов (1/30), но при этом 28% антиретровирусных препаратов (9/32) и 23% противомалярийных препаратов (6/26) не имели монографий.

**Вывод:**

По-видимому, для многих так называемых основных лекарств нет общедоступных стандартов. Для устранения этого недостатка в мировом сообществе специалистов здравоохранения необходимо более активное сотрудничество по данному вопросу.****

## Introduction

Despite the regulation of medicines, by the relevant regulatory authorities working in partnership with civil society, customs, health departments and law-enforcement agencies, exposure to substandard and/or falsified medicines remains an all-too-common experience, especially for patients in low- and middle-income countries.^1^

To ensure that a medicine meets the relevant, internationally-accepted quality standards, regulators perform analytical testing of medicines, along with other regulatory functions, such as inspection, pharmacovigilance and registration. In general, the quality standards used are those given in a pharmacopeial monograph on the medicine of interest, although not all medicines are covered by such monographs. A pharmacopeial monograph provides detailed parameters that are used to determine whether a medicine meets key quality attributes and can be marketed legally in any given country.^2^ Although, ideally, every generic medicine sold in a country should be covered by a pharmacopeial monograph, many countries around the world do not have their own national pharmacopeias. In addition, the national pharmacopeias that do exist may not have the capacity or resources to develop monographs covering all of the medicines needed in their countries. Subsequently, many countries legally adopt the monographs of several of the larger pharmacopeias, such as the British, European, Japanese and/or United States Pharmacopeias. However, these larger pharmacopeias tend to be focused on the medicines commonly used in a particular high-income country or area of the world and may fail to cover some other medicines that are commonly used in developing countries. The World Health Organization (WHO) has attempted to address this problem by publishing the International Pharmacopeia, which gives priority to medicines of major, global, public health importance, focuses on medicines important for WHO health programmes around the world and develops standards for medicines that are not covered by monographs in other pharmacopeias.^3^

WHO has also published many editions of its Model List of Essential Medicines.^4^ The 20th edition of the Model List, published in March 2017, lists 669 so-called essential medicines, that is, medicines that satisfy the priority health care needs of the population, divided into 30 sections and 109 sub-sections.^5^ In 2017 we decided to check which of the essential medicines on the Model List were covered by pharmacopeial monographs. We had three main aims: to raise awareness about the importance of public pharmaceutical standards; identify if and where current pharmacopeias are falling short; and foster collaboration among the various pharmacopeias, to prioritize, develop, and make available standards for those key medicines that currently have either no monograph or an incomplete monograph.

## Methods

In August 2017 we checked to see which medicines in the 20th edition of the Model List were covered by monographs in any of eight of the larger pharmacopeias. The pharmacopeias investigated were: the fifth edition of the Brazilian Pharmacopeia, including the 2016 supplement;^6^ the 2017 version of the British Pharmacopeia;^7^ the eighth edition of the Indian Pharmacopeia Commission, published in 2015;^8^ the sixth edition of the International Pharmacopeia, published in 2016;^3^ the 17th edition of the Japanese Pharmacopeia, published in 2016;^9^ the 11th edition of the Mexican Pharmacopeia,^10^ including the 2014,^11^ 2015^12^ and 2016^13^ supplements; the 2015 version of the Pharmacopeia of the People’s Republic of China;^14^ and the 2017 version of the United States Pharmacopeia.^15^

Ordinarily, the attributes that define the quality of a medicine are, at least, the identity of the active pharmaceutical ingredient, the content of the active pharmaceutical ingredient and the performance of the drug product, e.g. the disintegration and dissolution. In evaluating the completeness of a pharmacopeial monograph, we focused on those factors relevant to analytical test procedures and ignored labelling, packaging and storage requirements. To be considered complete, a monograph had to define an identification procedure, an assay procedure to measure the content of the active pharmaceutical ingredient, an impurities procedure and, if relevant, a dissolution procedure to determine the rate of release of the drug substance or active ingredient from a drug product. We considered a monograph for a form of drug product that does not commonly have a dissolution procedure, e.g. an injection, complete if it contained identity, assay and impurities procedures and another dosage-form-specific test (Table 1) and incomplete if it lacked a dosage-form-specific test. In checking the eight pharmacopeias, we made no attempt to review or evaluate the assay and test procedures that were described. In our data analysis, we considered several dosage forms of a single active ingredient to be interchangeable (Table 1). Below, as in the Model List, we separate two active pharmaceutical ingredients in a fixed-dose combination product with a plus sign, e.g. artesunate + amodiaquine.

**Table 1:** Dosage forms and corresponding quality-control procedures considered in a review of pharmacopeial monographs, 2017

Main category of dosage form	Equivalent dosage forms	Common quality-control procedures
Injection	Powder for injection, solutions for infusions, parenteral formulations	Identity, assay, impurities, particulate matter, sterility, bacterial endotoxins
Oral liquid	Oral solution, oral suspension, oral drops, powder for oral solution, powder for oral suspension, syrup	Identity, assay, impurities
Eye drops	Ophthalmic suspension, ophthalmic solution	Identity, assay, impurities
Solid oral dosage forms	Any form of tablet, including scored and unscored tablets, capsules and lozenges	Identity, assay, impurities, dissolution, disintegration

We investigated the British, International, Japanese and United States Pharmacopeias as the four internationally recognized and most frequently used compendia for drug products. We added the Brazilian, Chinese, Indian and Mexican Pharmacopeias as each represents a major hub for the manufacture of pharmaceutical products and is a large pharmacopeia in terms of the number of drug-product monographs.^16^ Six of the eight pharmacopeias we studied are published in English. The Brazilian and Mexican Pharmacopeias are published in Portuguese and Spanish, respectively. For these, we used Google Translate (Google LCC, Mountain View, United States of America) and the International Drug Name Database^17^ to facilitate translations of the medicines and procedures, where necessary. We chose not to investigate another large pharmacopeia, the European Pharmacopeia, because it focuses on drug substances rather than drug products. However, many of the drug-substance monographs in the British Pharmacopeia, which we did investigate, are identical to the drug-substance monographs in the European Pharmacopeia.

Although the 20th edition of Model List appears to include 765 medicines,^5^ 69 are listed at least twice in multiple sections of the Model List, either as equivalent dosage forms or in different sections of the Model List. For example, acetylsalicylic acid tablets are found in the antimigraine, antithrombotic, juvenile joint disease and non-opioid and non-steroidal anti-inflammatory medicine sections of the Model List. Our data analysis was confined to the 669 unique medicines on the Model List. All of the numbers and percentages we report were determined and calculated independently by two analysts, cross-checked and then confirmed by a reviewer.

Each analyst first reviewed the Model List and developed a database that was initially based on five fields: therapeutic category, medicine name, dosage form, section and sub-section. Subsequently, after reviewing all eight pharmacopeias we studied, each analyst added a sixth field to the database, in which the analyst indicated whether or not a medicine on the Model List was covered by a monograph in any of the eight pharmacopeias. The two databases were then checked against one another and consolidated for the data analysis.

## Results

**Fig. 1:**
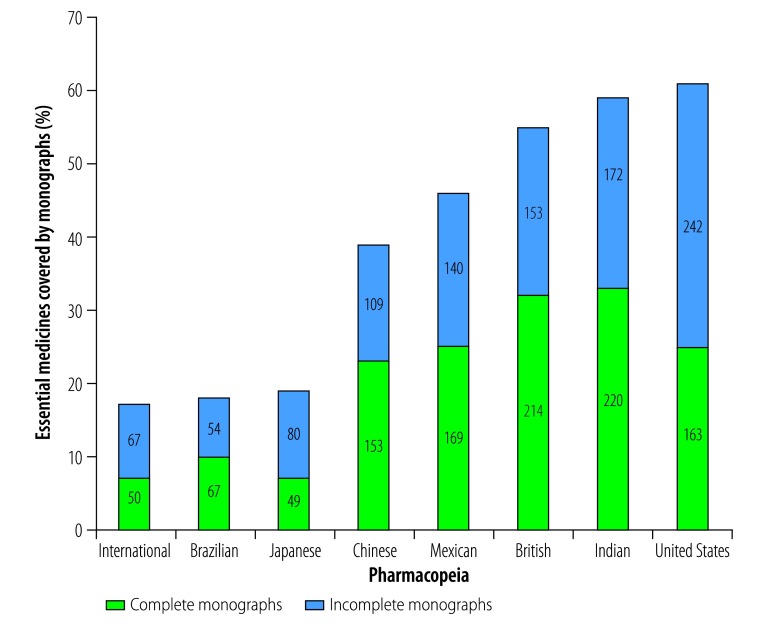
Numbers and percentages of 669 essential medicines covered by monographs in each of eight pharmacopeias, 2017

Notes: Medicines were considered essential if listed on the 20th edition of the World Health Organization’s Model List of Essential Medicines.^5^ Eight pharmacopeias were searched for monographs on each medicine. A monograph was only considered complete if it gave details of identity, assay and impurities procedures and either a dissolution assay or other dosage-form-specific test. Although percentages are indicated by the heights of the bars, the numbers within the plot area indicate absolute numbers of monographs.

Across the eight compendia we studied, we found 2091 monographs for the 669 unique medicines on the Model List (Fig. 1). However, we only found complete monographs for 340 (51%) of the medicines. The other medicines were either covered by incomplete monographs (230; 34%) or were not covered by any of the eight pharmacopeias that we studied (99; 15%).

Only 519 (78%) of the 669 medicines on the Model List were covered by the British, International, Japanese and/or United States Pharmacopeias, either as complete monographs (192; 29%) or incomplete monographs (327; 49%).

Only eight medicines on the Model List had a monograph in all eight of the pharmacopeias that we studied. Seven of these medicines, that is, acyclovir tablets, ampicillin powder for injection, clindamycin capsules, isoniazid tablets, metronidazole tablets, solid oral-dosage forms of rifampicin and streptomycin injection, fell within the anti-infective section of the Model List.

**Fig. 2:**
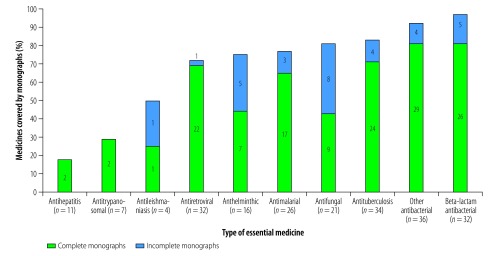
Numbers and percentages of essential anti-infective medicines covered by at least one monograph in eight pharmacopeias, 2017

Notes: Medicines were considered essential if listed on the 20th edition of the World Health Organization’s Model List of Essential Medicines.^5^ Eight pharmacopeias, i.e. the Brazilian, British, Indian, International, Japanese, Mexican and United States Pharmacopeias and the Pharmacopeia of the People’s Republic of China, were searched for monographs on each medicine. A monograph was only considered complete if it gave details of identity, assay and impurities procedures and either a dissolution assay or other dosage-form-specific test. Although percentages are indicated by the heights of the bars, the numbers within the plot area indicate absolute numbers of monographs.

**Fig. 3:**
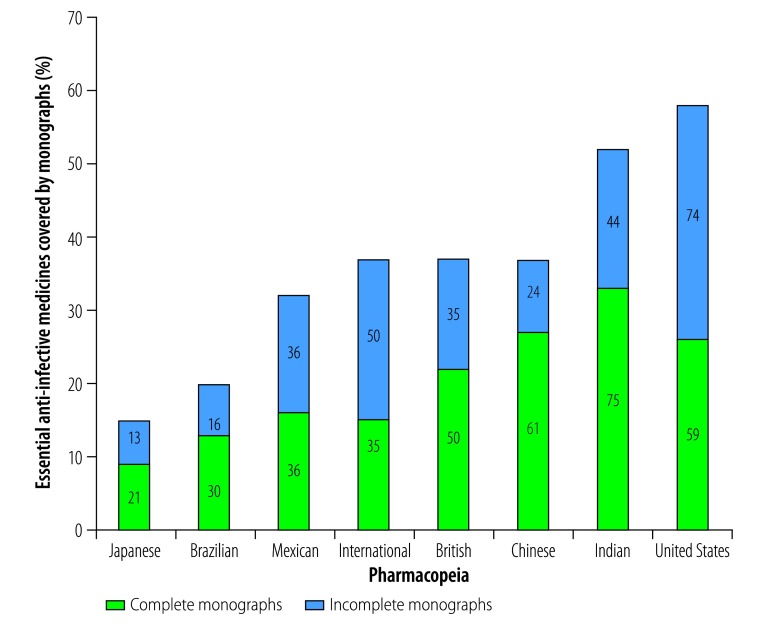
Numbers and percentages of 219 essential anti-infective medicines covered by monographs in each of eight pharmacopeias, 2017

Notes: Medicines were considered essential if listed on the 20th edition of the World Health Organization’s Model List of Essential Medicines.^5^ Eight pharmacopeias were searched for monographs on each medicine. A monograph was only considered complete if it gave details of identity, assay and impurities procedures and either a dissolution assay or other dosage-form-specific test. Although percentages are indicated by the heights of the bars, the numbers within the plot area indicate absolute numbers of monographs.

Due to of the increasing interest in and the public health importance of antimicrobial resistance, the large number of anti-infective medicines on the Model List and the high priority given to several infective diseases, e.g. acquired immunodeficiency syndrome (AIDS), malaria and tuberculosis, we conducted an additional analysis that was focused on the anti-infective section of the Model List. Most of the monographs on anti-infective medicines that we found were complete (Fig. 2). Overall, 97% (31/32) of the β-lactam antibacterials on the Model List were covered by at least one monograph in the pharmacopeias that we studied and 81% (26/31) of the β-lactam monographs were complete. In contrast, we found few monographs covering antihepatitis (18%; 2/11), antileishmaniasis (50%; 2/4), antitrypanosomal (29%; 2/7), antimalarial (77%; 20/26), antiretroviral (72%; 23/32) and antituberculosis (82%; 28/34) medicines on the Model List. Fig. 3 shows the numbers of complete and incomplete monographs available, from each pharmacopeia that we studied, for all of the 219 anti-infective medicines on the Model List.

To allow us to compare the availability of monographs for medicines against communicable diseases with that of monographs for medicines against noncommunicable diseases, we also investigated monographs for medicines against cardiovascular disease. We failed to find a monograph for just one (3%) of the 30 cardiovascular medicines on the Model List: hydrochlorothiazide oral solution. In addition, only two of the other cardiovascular medicines on the Model List were covered by monographs that were incomplete: glyceryl trinitrate tablets and methyldopa tablets. Similarly, we found complete monographs for all five of the antidiabetes medicines and for all seven of the non-steroidal and anti-inflammatory medicines on the Model List. In contrast, we failed to find a monograph for 49 (21%) of the 229 anti-infective medicines on the Model List (Table 2) while a further 39 (17%) such medicines were only covered by monographs that were incomplete.

**Table 2:** Essential anti-infective medicines that were not covered by monographs in the Brazilian, British, Indian, International, Japanese, Mexican or United States Pharmacopeias or the Pharmacopeia of the People’s Republic of China, 2017

Section of WHO’s Model List^a^	Medicine	Dosage form
6.1 anthelminthics	Oxamniquine	Capsule
	Oxamniquine	Oral liquid
	Pyrantel (as pamoate)	Tablet (chewable)
	Triclabendazole	Tablet
6.2 antibacterials	Bedaquiline	Tablet
	Ceftaroline	Powder for injection
	Ciprofloxacin (as hyclate)	Solution for IV infusion
	Colistin	Powder for injection
	Daptomycin	Powder for injection
	Linezolid	Injection for IV administration
	Linezolid	Powder for oral liquid
	Para-aminosalicylic acid	Granules
	Pyrazinamide	Tablet (dispersible)
	Rifapentine	Tablet
6.3 antifungals	Amphotericin B (liposomal complex)	Powder for injection
	Itraconazole	Oral liquid
	Voriconazole	Powder for injection
	Voriconazole	Powder for oral liquid
6.4 antivirals	Abacavir (as sulfate)	Tablet (dispersible)
	Abacavir + lamivudine (as sulfate)	Tablet (dispersible)
	Atazanavir + ritonavir	Tablet (heat stable)
	Daclatasvir (as hydrochloride)	Tablet
	Darunavir	Tablet
	Dasabuvir	Tablet
	Dolutegravir	Tablet
	Entecavir	Oral liquid
	Isoniazid + pyridoxine + sulfamethoxazole + trimethoprim	Tablet (scored)
	Ledipasvir + sofosbuvir	Tablet
	Ombitasvir + paritaprevir + ritonavir	Tablet
	Pegylated interferon α (2a or 2b)	Vial or prefilled syringe
	Raltegravir	Tablet
	Raltegravir	Tablet (chewable)
	Simeprevir	Capsule
	Sofosbuvir	Tablet
	Sofosbuvir + velpatasvir	Tablet
	zidovudine	Tablet (dispersible)
6.5 antiprotozoals	Artemether + lumefantrine	Tablet (dispersible)
	Artesunate	Rectal dosage form
	Artesunate + amodiaquine	Tablet
	Artesunate + mefloquine	Tablet
	Artesunate + pyronaridine tetraphosphate	Tablet
	Artesunate + pyronaridine tetraphosphate	Granules
	Benznidazole	Tablet
	Eflornithine (as hydrochloride)	Injection
	Miltefosine	Solid oral dosage form
	Nifurtimox	Tablet
	Paromomycin (as sulfate)	Solution for IM injection
	Pentamidine (as isethionate)	Tablet
	Suramin sodium	Powder for injection

IM: intramuscular; IV: intravenous; WHO: World Health Organization.

^a^ WHO’s Model List of Essential Medicines.

## Discussion

The availability of monographs providing public standards for medicines allows official medicine-control laboratories, manufacturers and other relevant stakeholders to assure the quality of medicines, before they are passed to the general public. Our results of checking eight major pharmacopeias in 2017 indicate that monographs do not exist for more than one in every seven medicines on the 20th edition of the Model List. We also failed to find a complete monograph for an additional one-third of the medicines on the same edition of the Model List. In other words, we failed to find a complete monograph for almost half of all of the medicines on the Model List. Given that the medicines on the Model List should satisfy the priority health needs of the population,^18^ the absence of the standards needed to assure the quality of many medicines on the Model List is unacceptable. Of the medicines on the Model List for which we failed to find even an incomplete monograph, over half are anti-infective medicines that are used primarily to treat diseases that disproportionately afflict the developing world, for example malaria, tuberculosis and some of the so-called neglected tropical diseases.

The shortage of adequate pharmacopeial monographs that we have recorded may reflect the dependency of many pharmacopeias on donations from their local pharmaceutical industry and, also, geographical bias. Three of the world’s most influential and/or most used pharmacopeias, that is the British, Japanese and/or United States Pharmacopeias, primarily serve high-income member countries of the Organisation for Economic Co-operation and Development. The medicines used in these countries, and by extension, the monographs developed by these countries’ national pharmacopeias, tend to be tailored to the domestic disease burdens. In consequence, medicines associated with the prevention of substantial pathology in high-income counties tend to be well covered by the British, Japanese and/or United States Pharmacopeias whereas the corresponding coverage of medicines that are rarely needed in high-income countries is often relatively poor. Such trends probably explain why, in our study, we found complete monographs for almost all of the medicines on the Model List used against cardiovascular disease but for only two of the seven antitrypanosomal medicines.

We believe that there are three main factors that promote or discourage the development of complete pharmacopeial monographs: regulatory requirements; an over-dependence on standards created by drug manufacturers; and an absence of a global infrastructure for the promotion, revision and support of pharmacopeial monographs.

In any given country, the availability of a monograph on a particular drug product may be determined by myriad factors, one of which, logically, is whether or not the drug product involved is both needed and registered in the country. In general, if the product is registered in a country, the corresponding pharmacopeial monograph becomes the legally recognized documentary standard and subsequent generic medicines must comply with the standard submitted by the original registering manufacturer. Often, there is little commercial incentive for a manufacturer to register a drug in a country where that drug will not be used frequently, e.g. an antimalarial medicine in, the United Kingdom of Great Britain and Northern Ireland. In general, a national pharmacopeia is unlikely to publish monographs for products that are not registered in the pharmacopeia’s host country. The resultant shortfall in pharmacopeial monographs is a major challenge for all those working to increase the availability of public standards that help ensure the efficacy and safety of medicines, particularly those medicines of public health importance. Recognizing this limitation, the United States Pharmacopeia has, with the insight and support of other key stakeholders, launched a new section within its compendia entitled Global Health Monographs. The intention is that this new section, while not disrupting the United States Pharmacopeia’s domestic legal mandate and activities, supports the development of monographs and other regulatory activities for drug products that are not marketed in the United States.^19^

To encourage manufacturers to produce and market the medicines needed in developing countries, some local regulators do not require generic medicines to meet the specifications of a pharmacopeial monograph. If, in these circumstances, a recognized public monograph does not exist, a manufacturer may submit their own internal methods as part of the registration process. However, such methods may not have been verified by an independent body and there may be no chemical reference standard available.^2^ In addition, if appropriate legislative structures are not in place, subsequent manufacturers may not have to meet the specifications of the first-to-market manufacturer’s method and there may be no incentive for a manufacturer to collaborate with a pharmacopeia and turn an internal standard into a public pharmacopeial monograph.

The development of new analytical techniques and more robust methods has already led to substantial improvements in the quality of some pharmacopeial monographs, which have become more precise and specific. If there are to be more such improvements, there needs to be a global infrastructure for the proactive, focused development and modernization of monographs. The creation of that infrastructure could be facilitated through intra-pharmacopeial collaboration, industry–pharmacopeia partnerships or even regulatory work-sharing groups, such as the International Generic Drug Regulators Programme.^20^ It is noteworthy that, although we found that 49 of the anti-infective medicines on the Model List were not covered by monographs in the British, International, Japanese and/or United States Pharmacopeias, 14 of these medicines were covered by monographs in at least one of the other pharmacopeias we investigated. It would probably be a waste of resources for the British, International, Japanese and/or United States Pharmacopeias to develop monographs for these 14 medicines from scratch. Data sharing between pharmacopeias and the joint development of new monographs, particularly for those essential medicines for which no complete pharmacopeial monograph exists, needs to be encouraged. To begin, under the auspices of the International Meeting of World Pharmacopeias,^16^ the major pharmacopeias should share information related to their monograph pipelines proactively and subsequently develop a joint work plan to target key medicines. This would avoid duplication of efforts and ensure that essential medicines lacking monographs are given priority. Beyond collaboration between pharmacopeias, a more concerted partnership between the pharmacopeias and pharmaceutical industry needs to be established. This partnership should focus on the identification of all medicines on the Model List that lack complete monographs and have either received marketing authorization from a stringent regulatory authority or been prequalified by WHO, e.g. artemether + lumefantrine dispersible tablets or artesunate + amodiaquine tablets, and the subsequent development of new monographs and related chemical reference standards.

Our study has some limitations. While the eight pharmacopeias that we selected represent those most used around the world, they do not represent all of the pharmacopeias currently in use. Our findings apply only to a single time-point: August 2017. Over time, as new editions of the pharmacopeias that we studied are released, monographs will be added or deleted. In the future, more pharmacopeias need to be investigated. As we made no attempt to evaluate the quality of the procedures described in the monographs we investigated, we recommend a future study in which the relevance and quality of existing analytical procedures are reviewed, at least for a subsample of monographs for medicines on the Model List. Finally, many relevant treatment guidelines, published by WHO and national and other international and multilateral organizations, include medicines that do not appear on the 20th edition of the Model List. Future work could incorporate such medicines.

In conclusion, the 20th edition of WHO’s Model List of Essential Medicines is based on medicines that have been identified by the global health community as those of critical public health importance. They are medicines that enable many sufferers of chronic diseases, such as AIDS, hepatitis C and tuberculosis, to live fulfilling and productive lives and they also help prevent maternal and child mortality. While most of the medicines on the Model List are covered by pharmacopeial monographs, key tools enabling regulatory authorities to detect substandard and falsified medicines – many are not. Efforts to resolve this shortfall need to be promoted. We wish to engender the collaborative development of, and ensure the availability of, public standards to assure the quality of medicines around the world.
